# Particulate matter-induced senescence of skin keratinocytes involves oxidative stress-dependent epigenetic modifications

**DOI:** 10.1038/s12276-019-0305-4

**Published:** 2019-09-24

**Authors:** Yea Seong Ryu, Kyoung Ah Kang, Mei Jing Piao, Mee Jung Ahn, Joo Mi Yi, Guillaume Bossis, Young-Min Hyun, Chang Ook Park, Jin Won Hyun

**Affiliations:** 1 Jeju National University School of Medicine and Jeju Research Center for Natural Medicine, Jeju, 63243 Republic of Korea; 2 Laboratory of Veterinary Anatomy, College of Veterinary Medicine, Jeju National University, Jeju, 63243 Republic of Korea; 3 Department of Microbiology and Immunology, Inje University College of Medicine, Busan, 47392 Republic of Korea; 4 Institut de Génétique Moléculaire de Montpellier, University of Montpellier, CNRS, Montpellier, France; 5 Department of Anatomy, Yonsei University College of Medicine, Seoul, 03722 Republic of Korea; 6 Department of Dermatology & Cutaneous Biology Research Institute, Yonsei University College of Medicine, Seoul, 03722 Republic of Korea

**Keywords:** Cell biology, Biochemistry

## Abstract

Ambient air particulate matter (PM) induces senescence in human skin cells. However, the underlying mechanisms remain largely unknown. We investigated how epigenetic regulatory mechanisms participate in cellular senescence induced by PM with a diameter <2.5 (PM_2.5_) in human keratinocytes and mouse skin tissues. PM_2.5_-treated cells exhibited characteristics of cellular senescence. PM_2.5_ induced a decrease in DNA methyltransferase (DNMT) expression and an increase in DNA demethylase (ten–eleven translocation; TET) expression, leading to hypomethylation of the *p16*^*INK4A*^ promoter region. In addition, PM_2.5_ led to a decrease in polycomb EZH2 histone methyltransferase expression, whereas the expression of the epigenetic transcriptional activator MLL1 increased. Furthermore, binding of DNMT1, DNMT3B, and EZH2 to the promoter region of *p16*^*INK4A*^ decreased in PM_2.5_-treated keratinocytes, whereas TET1 and MLL1 binding increased, leading to decreased histone H3 lysine 27 trimethylation (H3K27Me3) and increased H3K4Me3 in the promoter of *p16*^*INK4A*^. PM_2.5_-induced senescence involved aryl hydrocarbon receptor (AhR)-induced reactive oxygen species (ROS) production. ROS scavenging dampened PM_2.5_-induced cellular senescence through regulation of DNA and histone methylation. Altogether, our work shows that skin senescence induced by environmental PM_2.5_ occurs through ROS-dependent the epigenetic modification of senescence-associated gene expression. Our findings provide information for the design of preventive and therapeutic strategies against skin senescence, particularly in light of the increasing problem of PM_2.5_ exposure due to air pollution.

## Skin damage: counteracting the effects of air pollution

Fine particulate matter in polluted air damages skin cells by increasing oxidative stress and the expression of a protein that stops cell division. Jin Won Hyun and colleagues at Jeju National University School of Medicine, South Korea, show that exposure to particulate matter emitted by diesel engines causes skin cell senescence. In previous studies, they reported that particulate matter exposure led to the generation of reactive oxygen species (ROS). This study shows that ROS triggers changes in DNA and histone-modifying enzymes that remove suppressive chemical markers from the gene encoding a protein associated with senescence, p16^INK4^. Interestingly, treatment with the antioxidant N-acetylcysteine reduced both oxidative stress and p16^INK4^ expression. These findings could guide the development of new skin-care products that prevent damage due to air pollution.

## Introduction

Urban air pollution continues to be a serious health issue. Air pollution comprises a large proportion of particulate matter (PM) generated by coal combustion and diesel automobile exhaust^1^. Respiratory and cardiovascular diseases caused by PM are widely recognized. In addition, the skin, as the largest organ and outer layer of the human body, is constantly exposed to external stimuli and is thus a direct target of PM. Polycyclic aromatic hydrocarbons (PAHs) attached to PM represent important active agents that induce skin allergy, inflammatory dermatitis, eczema, and senescence^2–5^. PAHs such as benzo[*a*]pyrene induce toxicity in various cell types by activating aryl hydrocarbon receptor (AhR) signaling. AhR is a ligand-activated transcription factor. Upon ligand binding, AhR translocates from the cytosol to the nucleus and interacts with the AhR nuclear translocator to initiate transcription^6^. AhR activation can lead to oxidative stress as a result of excess generation of reactive oxygen species (ROS)^7^. These ROS and free radicals play a major role in skin senescence, inflammatory skin diseases, and carcinogenesis^8–10^.

Senescent cells remain metabolically active but cannot express genes required for cell proliferation. Senescent cells are typically characterized by a large and flat morphology, β-galactosidase activity, and senescence-associated heterochromatin foci (SAHF)^11^. The tumor suppressor protein p16^INK4A^ is a key regulator of senescence and arrests the cell cycle by functioning as a specific inhibitor of cyclin-dependent kinases (CDKs) 4 and 6^12^. In addition, the removal of p16^INK4A^-positive senescent cells has been found to delay senescence-associated disorders^13^.

Mammalian gene expression is tightly modulated by distinct epigenetic modifications, including DNA methylation and histone modifications. DNA methylation, which generally leads to transcriptional silencing, is one of the best-studied epigenetic modifications. DNA methylation is catalyzed by three DNA methyltransferases (DNMTs): DNMT1, DNMT3A, and DNMT3B, all of which add a methyl group to the C5 position of the cytosine ring of DNA, resulting in 5-methylcytosine (5-mC). This methylation process can be reversed by three DNA demethylases (ten–eleven translocations; TETs): TET1, TET2, and TET3. These enzymes can convert 5-mC to 5-hydroxymethylcytosine (5-hmC), 5-formylcytosine, and 5-carboxylcytosine, ultimately yielding cytosine^14^.

Expression of the p16^INK4a^ protein, encoded by the cyclin-dependent kinase inhibitor 2A gene^15^, is regulated by the DNA methylation of CpGs in its promoter, which reduces its expression^16,17^. In addition, the *p16*^*INK4A*^ locus is silenced in actively growing cells by histone H3 lysine 27 trimethylation (H3K27Me3), which is induced by EZH2, a histone methyltransferase and transcriptional suppressor^18^. When cells are exposed to cellular stress, H3K27Me3 levels at the locus decrease, resulting in p16^INK4A^ expression^19^. In contrast, MLL1 histone methyltransferase binds to the *p16*^*INK4A*^ locus and induces the H3K4Me3 mark, activating p16^INK4A^ transcription during replicative and premature senescence^20^.

Although many studies have led to a better understanding of the biochemical and cellular functions, as well as the epigenetic gene regulation of p16^INK4A^, the epigenetic regulation of p16^INK4A^ expression during PM_2.5_-induced skin senescence remains poorly understood. Here, we focused on the epigenetic regulation of p16^INK4A^ transcription and the molecular mechanisms by which they regulate p16^INK4A^ transcription during PM_2.5_-induced cellular senescence in human keratinocytes and mouse skin tissues.

## Materials and methods

### PM_2.5_ preparation

Diesel particulate matter (PM_2.5_), which is a standard diesel PM (SRM 1650b) according to the National Institute of Standards and Technology (NIST, USA), was purchased from Sigma-Aldrich (St. Louis, MO, USA). With a mean diameter of 0.18 µm, 1650b diesel PM was composed predominantly of PAHs and nitro-PAHs. Information on the certified mass fraction values of PAHs and nitro-PAHs in SRM 1650b is available in our recently published paper^21^. PM_2.5_ was dissolved in dimethyl sulfoxide (DMSO) at a concentration of 100%, and when applied to cells, the concentration of DMSO did not exceed 0.01%.

### Keratinocyte culture

The human keratinocyte cell lines HaCaT and HEK001 were obtained from the CLS Cell Lines Service (Eppelheim, Germany) and the American Type Culture Collection (Manassas, VA, USA), respectively. Normal human epidermal keratinocytes (NHEKs) derived from a foreskin specimen of a healthy male donor (18 years old) were obtained from Professor Jin Ho Chung (Seoul National University Hospital, Seoul, Republic of Korea). HaCaT cells were cultured in RPMI-1640 medium containing 10% heat-inactivated fetal calf serum at 37 °C in an incubator with a humidified atmosphere of 5% CO_2_. HEK001 cells were cultured in keratinocyte-SFM containing human recombinant EGF (Thermo Fisher Scientific, Inc., Waltham, MA, USA). Primary human HEKs were cultured in EpiLife serum-free medium with EpiLife growth supplement (Thermo Fisher Scientific, Inc.) and were used at the third or fourth passage. The procedures involving human subjects received prior approval from the Institutional Review Board of Seoul National University Hospital. The study was conducted according to the principles described in the Declaration of Helsinki.

### Murine experiment

HR-1 hairless male mice weighing 22–24 g (OrientBio, Gyeonggi-do, Republic of Korea) were maintained under a controlled temperature of 25–28 °C and a 12 h light/dark cycle and received a standard diet and water ad libitum. All experimental procedures were conducted in accordance with the guidelines for the care and use of laboratory animals of Jeju National University (Jeju, Republic of Korea) (permit number: 2017-0026). Mice were divided randomly into groups of four animals each. PM_2.5_ was dissolved in 100 μg/mL propylene glycol and 100 μM NAC, spread on a nonwoven polyethylene pad with an area of 1 cm^2^, and then applied to the mouse dorsal region for 7 consecutive days. The skin tissue was immediately dissected for histological and molecular analyses^22^.

### Detection of β-galactosidase activity

Cells were plated on coverslips, and SPiDER-βGal working solution (Dojindo Molecular Technologies, Inc., Rockville, MD, USA) was added to the plate for 15 min at 37 °C. The cells were mounted on microscope slides in mounting medium containing DAPI to label the nuclei. Images were collected using a Zeiss confocal microscope and Zeiss LSM 510 software.

### Cell viability and colony formation

To assess cell viability, cells were seeded in a 60-mm dish at a density of 0.1 × 10^5^ cells/mL and treated for 7 days with 50 μg/mL PM_2.5_ without medium change. The cells were then resuspended in a 0.4% trypan blue solution and incubated at room temperature for 15 min. Stained (dead) cells were counted under a light microscope. Next, to assess colony formation, cells were plated in 60-mm dishes at a density producing ~500 colonies per dish and treated with 50 μg/mL PM_2.5_ for 7 days without medium change. The resultant colonies were fixed with 75% ethanol and 25% acetic acid and stained with trypan blue. Colonies containing 50 or more cells were considered viable.

### DAPI staining for SAHF detection

Cells were mounted on microscope slides in mounting medium containing DAPI to label the nuclei. Images were collected using a confocal microscope and Zeiss LSM 510 software.

### Western blot analysis

Cell lysates (50 μg protein) were electrophoresed, transferred, and immunoblotted with specific antibodies. Anti-AhR, anti-TET1, and anti-TBP antibodies were purchased from Thermo Fisher Scientific, Inc.; the anti-DNMT1 antibody was from Abcam (Burlingame, CA, USA); the anti-DNMT3B antibody was from Santa Cruz Biotechnology (Santa Cruz, CA, USA); the anti-p16^INK4A^ and anti-EZH2 antibodies were from Cell Signaling Technology, Inc. (Danvers, MA, USA), and the anti-MLL1 antibody was purchased from Active Motif (Carlsbad, CA, USA). The membranes with bound primary antibodies (1:1000) were reacted with horseradish peroxidase (HRP)-conjugated secondary antibodies (Pierce, Rockland, IL, USA), and the protein bands were assessed by a western blotting detection kit (Amersham, Little Chalfont, Buckinghamshire, UK).

### Reverse transcription-PCR

The conditions for PCR using the forward primer 5′-GCAGCATGGAGCCTTCGGCT-3′ and the reverse primer 5′-TGCAGCACCACCAGCGTGTC-3′ for the amplification of *p16*^*INK4A*^ were as follows: 5 min at 94 °C for initial denaturation; 35 cycles of 1 min at 94 °C, 1 min at 56 °C, and 1 min at 72 °C; and final elongation for 7 min at 72 °C. PCR amplification was performed in a programmable thermal cycler.

### Immunofluorescence

After we fixed the cells with 4% paraformaldehyde for 30 min, they were permeabilized with PBS with 0.1% Triton X-100 for 2.5 min. The cells were treated with blocking medium (PBS containing 3% bovine serum albumin) for 1 h and incubated for 2 h with primary antibodies diluted in blocking medium. Primary antibody binding was assessed by an FITC- or Alexa594-bound secondary antibody (Santa Cruz Biotechnology) for 1 h. After the cells were mounted on microscope slides in mounting medium containing DAPI, they were imaged on a Zeiss confocal microscope using the LSM 510 program.

### Chromatin immunoprecipitation and quantitative PCR

TET1, DNMT1, DNMT3B, EZH2, H3K27Me3, MLL1, and H3K4Me3 antibodies and normal rabbit IgG were used for chromatin immunoprecipitation (ChIP) assays, which were assessed with a specific ChIP kit (Cell Signaling Technology). DNA (100 ng) recovered from the immune-precipitated complexes was subjected to quantitative PCR (qPCR). The primers for the *p16*^*INK4A*^ locus are described in Table 1^23^.

**Table 1:** Quantitative PCR primers used for ChIP along the *p16*^*INK4A*^ locus

Primer location	Primer set	Sequence (5'-3')
p16^INK4a^ (−0.6 kb)	A	CCCGTCCGTATTAAATAAACC
		GACTGCTCTCTCCTTCCC
p16^INK4a^ (−0.3 kb)	B	GGGCTCTCACAACTAGGAAAG
		GGGTGTTTGGTGTCATAGGG
p16^INK4a^ (+85 bp)	C	CCCCTTGCCTGGAAAGATAC
		AGCCCCTCCTCTTTCTTCCT
p16^INK4a^ (+0.5 kb)	D	CTGGAGGACGAAGTTTGC
		AGGAGGAGGTCTGTGATTAC
p16^INK4a^ (+1.5 kb)	E	GTGTTTCTCCTCTCCCTACTCC
		CCGGGTTCAAGCTGTTGGC
p16^INK4a^ (+5.6 kb)	F	ACCAAGACTTCGCTGACC
		CAAGGAGGACCATAATTCTACC

### Quantitative methylation-specific PCR and bisulfite sequencing

For quantitative methylation-specific PCR (qMSP) analysis, DNA (2 μg) was modified by bisulfite with a DNA methylation kit (Zymo Research, Orange, CA, USA), which converts nonmethylated cytosine to uracil and protects methylated cytosine. The methylation of gene promoter sites was assessed using qMSP primer pairs located close to the putative transcription start site in the 5′ CpG island. Bisulfite-treated DNA as the template and JumpStart REDTaq DNA polymerase (Sigma-Aldrich Co.) were used for amplification. For bisulfite sequencing, the PCR products were purified with a gel extraction kit (Qiagen GmbH, Hilden, Germany) and cloned using the TOPO TA vector system (Invitrogen, Carlsbad, CA, USA). Each clone was isolated and purified using a NucleoSpin plasmid isolation kit (Macherey-Nagel, Düren, Germany). Randomly selected positive clones (10–15 from each sample) were sequenced using the M13F primer, and the methylation status of each CpG dinucleotide was analyzed. For the quantification of p16^INK4A^ methylation, qMSP amplification was performed on bisulfite-treated samples and normalized based on Alu element amplification. qPCR was performed using a CFX96TM real-time system (BioRad, Hercules, CA, USA). MSP and bisulfite sequencing primers were from published reports^24,25^.

### Transfection of small interfering RNA

Control small interfering RNA (siRNA) (SS-1001), AhR sense (5′-UCAUGCAGCUGAUAUGCUU-3′) and antisense (5′-AAGCAUAUCAGCUGCAUGA-3′) siRNA, TET1#1 sense (5′-CAGUGUAACCAGCACAGUU-3′) and antisense (5′-AACUGUGCUGGUUACACUG-3′) siRNA, MLL1#1 sense (5′-GUCACAGUAGGUGAUCCUU-3′) and antisense (5′-AAGGAUCACCUACUGUGAC-3′) siRNA, and MLL1#2 sense (5′-CUAUUCUCGGGUCAUCAAU-3′) and antisense (5′-AUUGAUGACCCGAGAAUAG-3′) siRNA were purchased from Bioneer Corporation (Daejeon, Republic of Korea). TET1#2 siRNA (AM16708) was obtained from Invitrogen. Cells were transfected with Lipofectamine RNAiMax reagent (Invitrogen) in serum-free Opti-MEM. Then, 3 μL of lipofectamine was incubated with 1.5 mL of Opti-MEM for 5 min at room temperature. siRNA was added to the Opti-MEM-lipofectamine solution to a final concentration of 20 nM. The mixture was incubated for 5 min at room temperature. The transfection solution was incubated with the plates for 24 h.

### Immunoprecipitation

Cell lysates (100 μg protein) were reacted overnight at 4 °C with MLL, TET1, or IgG antibodies. Immune complexes were collected with protein A/G PLUS beads (Santa Cruz Biotechnology) overnight at 4 °C and washed with immunoprecipitation buffer. Equal amounts of the precipitates were separated by SDS-polyacrylamide gel electrophoresis, followed by western blot analysis with antibodies specific for MLL1 and TET1.

### Proximity ligation assay

The mouse/rabbit red starter Duolink kit (Sigma-Aldrich Co.) was used for this experiment. Cells were permeabilized according to the manufacturer’s instructions and incubated with goat anti-TET1 (1:100) or rabbit anti-MLL (1:1000) primary antibodies in fluorescence dilution buffer (5% fetal calf serum, 5% normal donkey serum, 2% bovine serum albumin in PBSCM, pH 7.6) for 2 h at room temperature and incubated with the proximity ligation assay (PLA) probes for 1 h at 37 °C in a humidified chamber. The ligation reaction was performed at 37 °C for 1 h in a humidified chamber. The cells were then incubated with the amplification mix for 2 h at 37 °C in a darkened humidified chamber, mounted using mounting medium, and imaged using a Zeiss confocal microscope and the LSM 510 program.

### Detection of intracellular ROS

The intracellular levels of ROS were evaluated by spectrophotometric assay using an Amplex® Red kit (Invitrogen). Briefly, 0.1 × 10^5^ cells/mL were seeded in a six-well plate. After 16 h of culture, the cells were treated with PM_2.5_. The cell pellet was resuspended in buffer (20 mM HEPES, 10 mM KCl, 1.5 mM MgCl_2_, 1 mM EDTA, 1 mM EGTA, 1 mM DTT, 0.1 mM PMSF, and protease inhibitors supplemented with 250 mM sucrose), and the cells were disrupted with a Dounce homogenizer. The reaction in the cell lysate was started with Amplex® Red solution prepared according to the manufacturer’s instructions (Amplex® Red reagent, 10-acetyl-3,7-dihydroxyphenoxazine in DMSO and 0.2 U/mL HRP). After incubation at 37 °C and 5% CO_2_ for 30 min, the absorbance at 560 nm was measured with a plate reader. To quantify ROS production, a standard curve for H_2_O_2_ was established in parallel. The results were expressed as H_2_O_2_ nmol released in 30 min per μg of protein.

### Histological analysis

After skin sections (5 µm) were stained with hematoxylin and eosin, the thickness of the epidermis (from the stratum basale to the stratum corneum) was measured in ten randomly chosen fields taken from three representative sections per group, followed by microscopic evaluation at X100 optical magnification with a digital camera. Immunohistochemistry was performed using an ABC Elite kit (Vector Labs). The primary antibodies, including antikeratin 10 (Invitrogen), antiproliferating cell nuclear antigen (PCNA) (Santa Cruz Biotechnology), anti-DNMT3B, anti-TET1, anti-EZH2, anti-MLL1, and anti-p16^INK4A^ antibodies, were incubated with the skin sections for 1 h. The sections were counterstained with hematoxylin before mounting.

### Statistical analysis

The results are presented as the mean ± the standard error of the mean (SEM). SigmaStat software v12 (SPSS, Chicago, IL, USA) was used for statistical analysis. The data were analyzed using one-way analysis of variance (ANOVA) with Tukey’s post hoc test. Differences were considered statistically significant at *p* < 0.05.

## Results

### PM_2.5_ induces keratinocyte senescence

Recently, we investigated the potential role of oxidative stress induced by PM_2.5_ by measuring ROS generation at various concentrations (25–100 μg/mL) of PM_2.5_. At 50 μg/mL PM_2.5_, ROS levels, oxidative stress-induced cell damage and inflammatory responses were clearly observed^21,26^. Therefore, PM_2.5_ at a concentration of 50 μg/mL was used as the optimal concentration for the induction of senescence in cells.

**Fig. 1:**
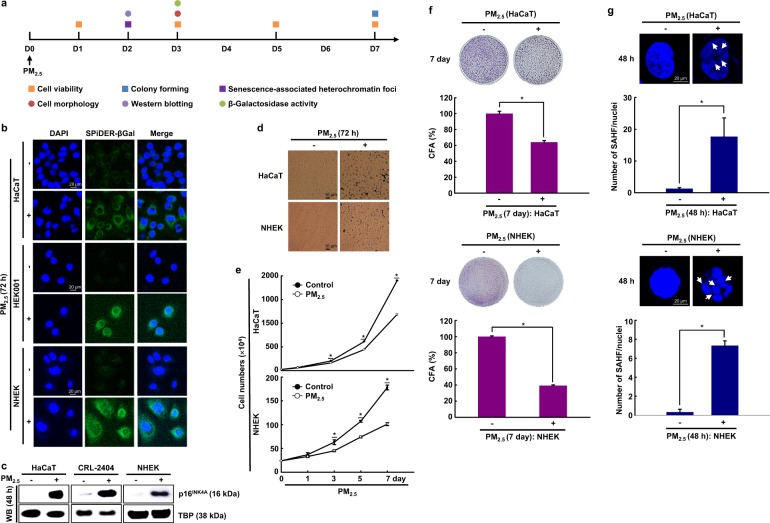
PM_2.5_-induced skin cellular senescence. **a** Human keratinocyte cell lines HaCaT and HEK001 and normal human epidermal keratinocytes (NHEKs) were each treated with 50 μg/mL PM_2.5_ and evaluated with various assays related to cellular senescence at the indicated time points. **b** Cells were stained for β-galactosidase activity using SPiDER-βGal working solution and DAPI. **c** Nuclear fractions were electrophoresed, and the senescence marker p16^INK4A^ was detected by western blot analysis with the corresponding antibodies. TBP is shown as a loading control. **d** Morphological changes accompanying senescence induced by PM_2.5_ were observed by microscopy. **e** Cell proliferation was evaluated using trypan blue dye exclusion assays. **f** Colonies were counted after trypan blue staining. **g** Cells were stained for SAHF structure, followed by DAPI staining. The arrows indicate SAHF in senescent cells induced by PM_2.5_. *Significantly different from untreated cells (*p* < 0.05)

The human keratinocyte cell lines HaCaT and HEK001 and NHEKs were treated with PM_2.5_ at 50 μg/mL, the optimal concentration for the induction of senescence in each of the cell types. PM_2.5_-treated cells were evaluated in various cellular senescence assays (Fig. 1a). PM_2.5_-treated cells exhibited β-galactosidase activity in the cytosol, a characteristic of cellular senescence, as evidenced by higher levels of green signal in PM_2.5_-treated cells than in untreated cells (Fig. 1b). Moreover, the expression of p16^INK4A^, a CDK inhibitor and senescence inducer, was considerably higher in PM_2.5_-treated cells than in the corresponding untreated cells (Fig. 1c). Furthermore, PM_2.5_-treated cells showed characteristics of cellular senescence, including an enlarged and flattened cell shape and irregular size (Fig. 1d), low proliferation (Fig. 1e), and decreased colony-forming ability (Fig. 1f). Chromatin in senescent cells undergoes large-scale rearrangement, forming dense nuclear domains called SAHF^26^. PM_2.5_-treated cells displayed significantly more SAHF-like chromatin foci in the nuclei than the controls (Fig. 1g).

### P16^INK4A^ is regulated epigenetically via DNA methylation during PM_2.5_-induced cellular senescence

**Fig. 2:**
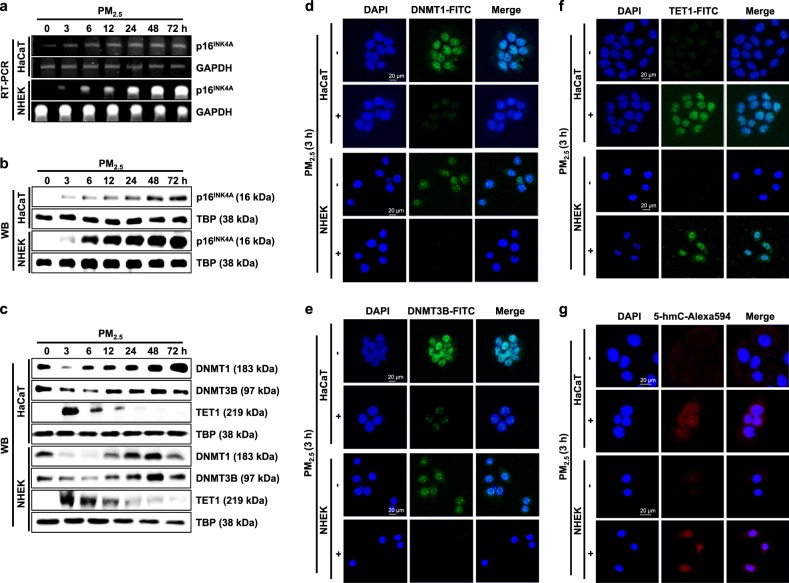
P16^INK4A^ transcriptional upregulation during PM_2.5_-induced cellular senescence correlates with DNA demethylation. The expression of p16^INK4A^ **a** mRNA and **b** protein at the indicated time points was assessed by RT-PCR and western blot assays, respectively. **c** Nuclear fractions were electrophoresed, and DNMT1, DNMT3B, and TET1 were detected by western blotting with the corresponding antibodies. TBP is shown as a loading control. The nuclear location of **d** DNMT1, **e** DNMT3B, and **f** TET1 was determined by confocal microscopy after FITC labeling with the corresponding antibody. **g** TET1 activity was detected by confocal microscopy after Alexa594 labeling with a 5-hmC antibody

**Fig. 3:**
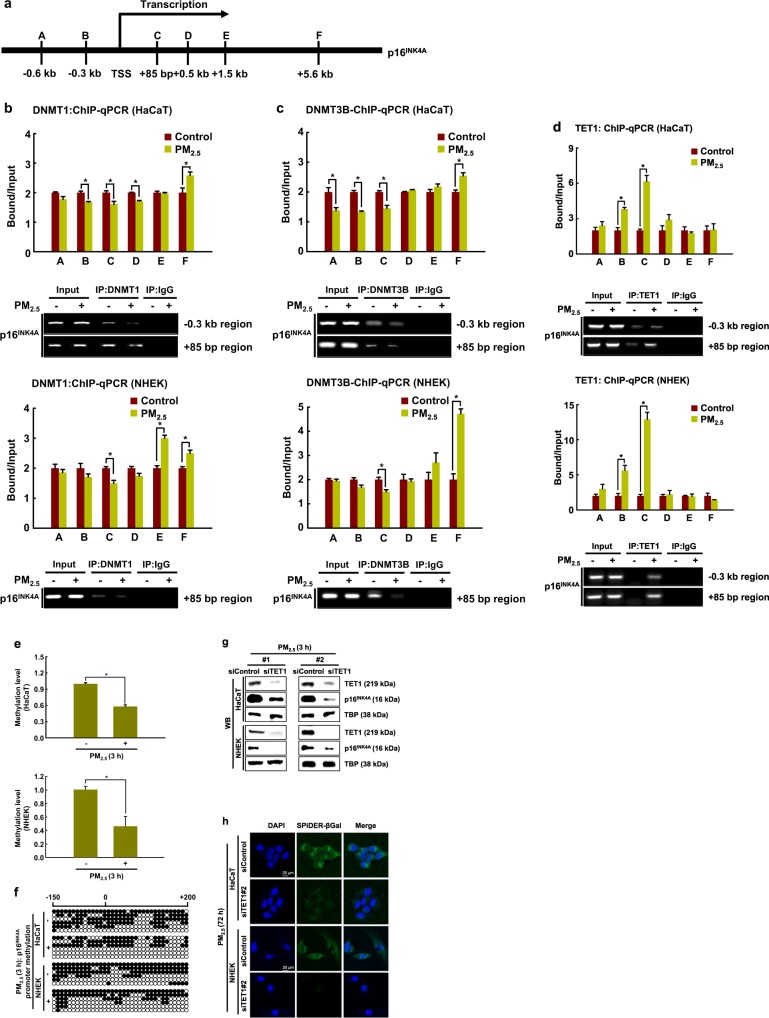
TET1 binds to and demethylates the *p16*^*INK4A*^ locus in response to PM_2.5_. **a** ChIP-qPCR was performed using primer sets specific for the regions indicated by A (−0.6 kb) to F (+5.6 kb) along the p16^INK4A^ locus. ChIP using antibodies against **b** DNMT1, **c** DNMT3B, and **d** TET1 were analyzed by qPCR. **e** Quantitative methylation levels of the *p16*^*INK4A*^ promoter region normalized to the Alu element. **f** Bisulfite sequencing analysis of the *p16*^*INK4A*^ promoter region. The black circles represent methylated cytosine resides; the white circles represent unmethylated cytosine residues. Cells were transfected with TET1 siRNA and incubated for 24 h. **g** TET1 and p16^INK4A^ were detected by western blotting with the corresponding antibodies. **h** β-Galactosidase activity was assessed by confocal microscopy after staining with SPiDER-βGal working solution and DAPI. *Significantly different from untreated cells (*p* < 0.05)

The mRNA expression of p16^INK4A^, a senescence inducer, was upregulated in PM_2.5_-treated cells from 3 h onwards, continuing up to 72 h (Fig. 2a). In agreement with the RT-PCR results, western blotting also revealed strong induction of the p16^INK4A^ protein (Fig. 2b). To investigate whether epigenetic DNA methylation could be involved in PM_2.5_-induced p16^INK4A^ expression, we measured the expression of DNA methylation-related proteins. As shown in Fig. 2c, DNMT1 and DNMT3B expression decreased 3–12 h after PM_2.5_ treatment, whereas TET1 expression increased transiently at 3–12 h after PM_2.5_ treatment. The decrease in the expression of DNMT1 and DNMT3B and the increase in TET1 observed at 3 h after treatment by western blot analysis was also confirmed by immunofluorescence (Fig. 2d–f). Concomitant with the PM_2.5_-induced increase in TET1 and decrease in DNMT expression, we observed increased conversion of 5-mC to 5-hmC (Fig. 2g). These results suggest that these changes in DNMT1 and TET1 expression affect the transcription of senescence-associated genes in response to PM_2.5_. Using ChIP-qPCR analysis, we then assessed whether DNMT1, DNMT3B, and TET1 could directly bind to the *p16*^*INK4A*^ locus in HaCaT cells and NHEKs (Fig. 3a). Binding of DNMT1 and DNMT3B to the *p16*^*INK4A*^ promoter region occurred in the absence of PM_2.5_. This binding decreased in the proximal promoter regions of *p16*^*INK4A*^ in HaCaT cells and NHEKs following PM_2.5_ treatment (Fig. 3b, c). The loss of DNMT1 and DNMT3B induced by PM_2.5_ prompted us to investigate whether this is accompanied by the appearance of TET1. We found that concomitant with the decreases in DNMT1 and DNMT3B, TET1 strongly binds to the B and C regions of the *p16*^*INK4A*^ promoter region in PM_2.5_-treated HaCaT cells and NHEKs (Fig. 3d). Collectively, these findings suggest that PM_2.5_-mediated p16^INK4A^ expression involves the recruitment of the DNA demethylase TET and the release of the DNMT, which participates in the induction of senescence.

We next examined the methylation status of the *p16*^*INK4A*^ promoter using qMSP and bisulfite sequencing analysis. As shown in Fig. 3e, the DNA methylation of the *p16*^*INK4A*^ promoter region was significantly lower in PM_2.5_-treated cells than in control cells. We then used bisulfite sequencing to examine DNA methylation in the *p16*^*INK4A*^ promoter region before and after PM_2.5_ treatment. Interestingly, the promoter region (from −150 to +200 bp, including regions B and C for ChIP and 35 CpG sites of *p16*^*INK4A*^) showed lower methylation in PM_2.5_-treated cells than in control HaCaT cells (58% vs. 31%) and control NHEKs (55% vs. 43%) (Fig. 3f). Therefore, the change in the transcription of *p16*^*INK4A*^ in PM_2.5_-treated HaCaT cells and PM_2.5_-treated NHEKs is likely due to epigenetic regulation through promoter methylation.

Because the increase in TET expression and hypomethylation of *p16*^*INK4A*^ preceded the induction of p16^INK4A^ during PM_2.5_-induced cellular senescence, we investigated whether the expression of p16^INK4A^ depended on TET. TET1 siRNA reduced the expression of p16^INK4A^ in PM_2.5_-treated cells, leading to a decrease in PM_2.5_-induced senescence (Fig. 3g, h). Thus, TET1 markedly contributes to the transcriptional activation of the *p16*^*INK4A*^ locus, which participates in the induction of cellular senescence.

### P16^INK4A^ is regulated epigenetically via histone methylation in PM_2.5_-induced cellular senescence

**Fig. 4:**
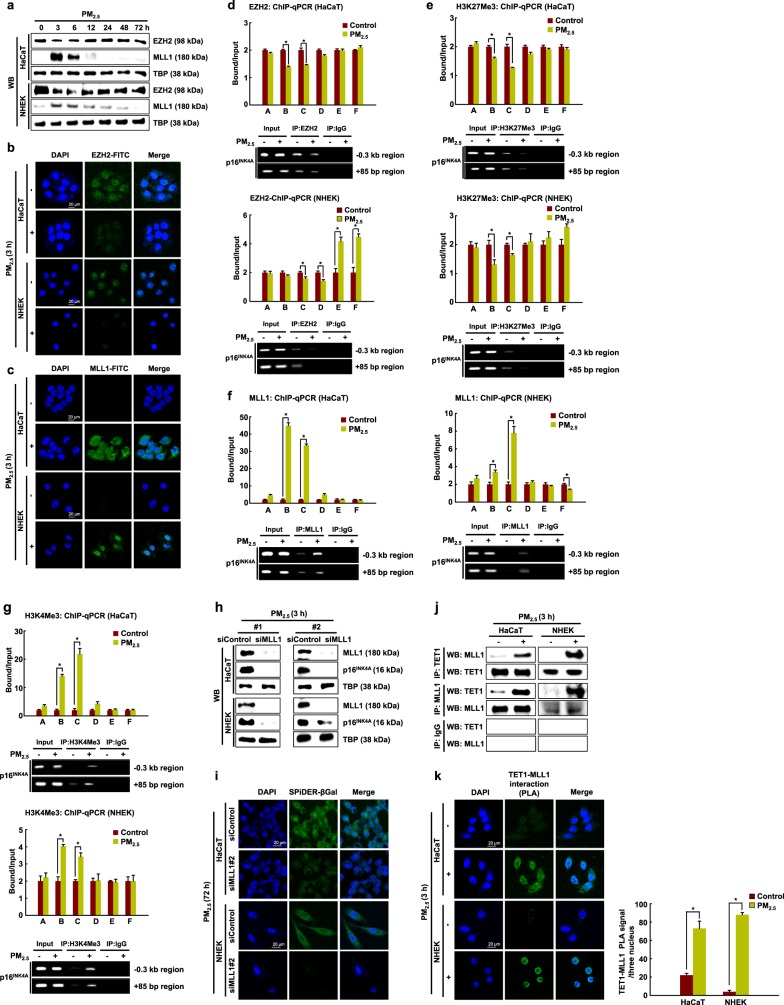
Senescence induced by PM_2.5_ is regulated via histone methylation of the *p16*^*INK4A*^ locus. **a** EZH2 and MLL1 were detected by western blotting with the corresponding antibodies. **b, c** The nuclear localization of EZH2 and MLL1 was detected by confocal microscopy after FITC labeling with the corresponding antibodies. ChIP products using antibodies directed against **d** EZH2, **e** H3K27Me3, **f** MLL1, and **g** H3K4Me3 were analyzed by qPCR. Cells were transfected with MLL1 siRNA and incubated for 24 h. **h** MLL1 and p16^INK4A^ were detected by western blotting with the corresponding antibodies. **i** β-Galactosidase activity was assessed by confocal microscopy after staining with SPiDER-βGal working solution and DAPI. The interaction between TET1 and MLL was examined by **j** immunoprecipitation and by **k** PLA. *Significantly different from untreated cells (*p* < 0.05)

To investigate whether epigenetic histone methylation could also be involved in PM_2.5_-induced p16^INK4A^ expression, we measured the expression of the histone methyltransferases EZH2 and MLL1 in response to PM_2.5_ by western blot analysis. As shown in Fig. 4a, the expression of EZH2, a component of the polycomb complex with H3K27 methyltransferase activity, decreased 3–12 h after PM_2.5_ treatment, whereas the expression of MLL1, which is a transcriptional activator and has H3K4 methyltransferase activity, increased transiently at 3–12 h after PM_2.5_ treatment. The decrease in EZH2 and the increase in MLL1 expression were confirmed by immunofluorescence at 3 h after PM_2.5_ treatment (Fig. 4b, c). We then wondered whether these changes in EZH2 and MLL1 could participate in the induction of the *p16*^*INK4A*^ locus in response to PM_2.5_. First, we assessed whether the silencer EZH2 occupies the *p16*^*INK4A*^ locus by ChIP-qPCR analysis. We observed the binding of EZH2 and the presence of H3K27Me3 in the *p16*^*INK4A*^ promoter region in the absence of PM_2.5_. The binding of EZH2 to the B and C regions of the *p16*^*INK4A*^ promoter in HaCaT cells and to the C and D regions in NHEKs significantly decreased upon PM_2.5_ treatment in HaCaT cells and NHEKs (Fig. 4d) and was linked to a decrease in H3K27Me3 at the same positions (Fig. 4e). The loss of EZH2 and the H3K27Me3 suppressive histone mark induced by PM_2.5_ prompted us to investigate whether this is accompanied by the appearance of the MLL1 activator and H3K4Me3-positive histone mark. We found that concomitant with the decrease in EZH2 and H3K27Me3, the active MLL1 and H3K4Me3 mark strongly increased in the B and C regions of the *p16*^*INK4A*^ promoter in PM_2.5_-treated HaCaT cells and NHEKs (Fig. 4f, g). Collectively, our results show that during PM_2.5_-mediated p16^INK4A^ expression, the transcriptional activator MLL1 can replace the transcriptional silencer EZH2 in its proximal promoter.

Because the increase in MLL expression precedes the induction of p16^INK4A^ during PM_2.5_-induced cellular senescence, we examined whether the expression of p16^INK4A^ depended on MLL1. MLL1 siRNA reduced the expression of p16^INK4A^ in cells treated with PM_2.5_ and limited the induction of cellular senescence (Fig. 4h, i). Thus, MLL significantly contributes to the transcriptional activation of the *p16*^*INK4A*^ locus. We then evaluated whether TET1 interacts with MLL in response to PM_2.5_. Using both immunoprecipitation and PLA, we showed that the interaction between TET1 and MLL was greater in PM_2.5_-treated cells than in untreated cells (Fig. 4j, k).

### PM_2.5_-induced cellular senescence is associated with oxidative stress via AhR-ROS signaling

**Fig. 5:**
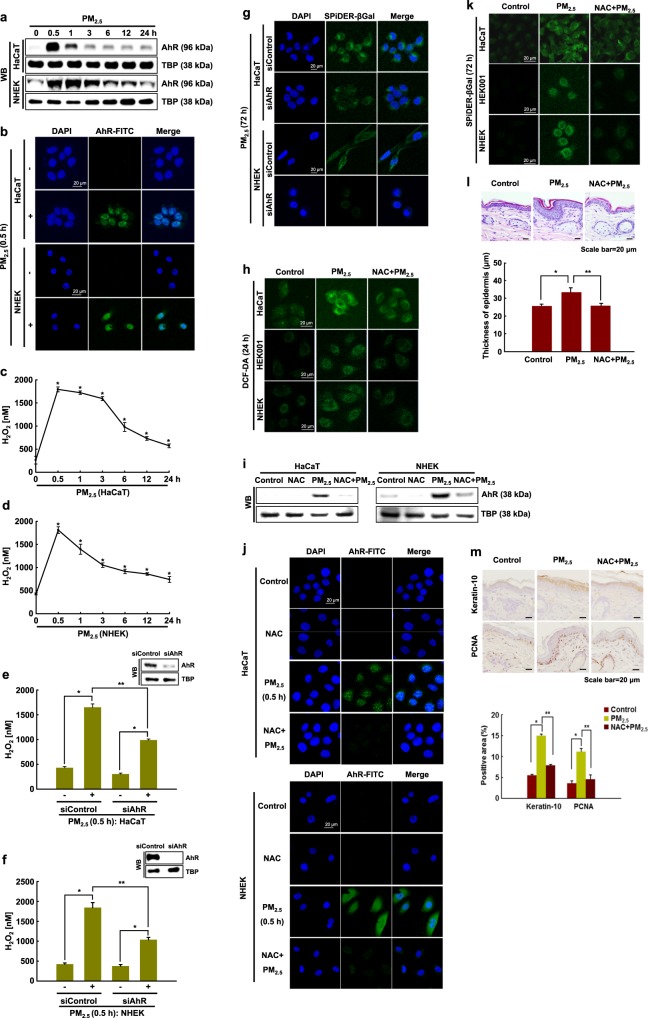
PM_2.5_-induced senescence involves the AhR-ROS signaling pathway. **a** Nuclear AhR in HaCaT cells and NHEKs was detected by western blotting with the corresponding antibody. **b** Nuclear AhR in HaCaT cells and NHEKs was detected by confocal microscopy after FITC labeling with an AhR antibody. Quantification of ROS production in **c** HaCaT cells and **d** NHEKs by the Amplex^®^ Red kit. *Significantly different from untreated cells (*p* < 0.05). Cells were transfected with AhR siRNA and incubated for 24 h, and ROS production was quantified in **e** HaCaT cells and **f** NHEKs. *Significantly different from untreated cells (*p* < 0.05). **g** β-Galactosidase activity was assessed by confocal microscopy after staining with SPiDER-βGal and DAPI. **h** The ROS level was assessed by confocal microscopy after DCF-DA staining. Nuclear AhR was detected by **i** western blotting and **j** confocal microscopy. **k** β-Galactosidase activity was assessed by confocal microscopy after staining with SPiDER-βGal. **l** Skin sections were assessed to determine the thickness of the mouse skin epidermis. **m** Skin sections were stained for keratin-10 and PCNA and counterstained with hematoxylin. *Significantly different from untreated cells (*p* < 0.05); **significantly different from PM_2.5_-treated cells (*p* < 0.05)

PAHs interact directly with AhR, promoting AhR translocation into the nucleus and leading to ROS production^27^. We thus hypothesized that PM_2.5_-induced cellular senescence might occur via the AhR–ROS signaling pathway. Interestingly, immunoblotting data showed that the nuclear accumulation of AhR in PM_2.5_-treated cells occurred at 0.5 h and continued until 24 h (Fig. 5a). The nuclear accumulation of AhR was confirmed by immunofluorescence at 0.5 h (Fig. 5b). In response to PM_2.5_, the ROS level increased from 0.5 h, and the increase continued until 24 h in HaCaT cells and NHEKs, concomitant with AhR expression (Fig. 5c, d). To further identify the role of AhR in PM_2.5_-induced skin cellular senescence, we used siRNA against AhR. AhR siRNA transfection attenuated the increase in ROS at 0.5 h after PM_2.5_ treatment in HaCaT cells and NHEKs (Fig. 5e, f) and attenuated the increase in β-galactosidase activity, a classic marker of senescence (Fig. 5g). In addition to changes in gene expression and metabolic control during the cellular senescence process, the senescence rate is associated with the production of high levels of ROS^28^. PM_2.5_-treated HaCaT and HEK001 cells and NHEKs showed significantly higher intracellular ROS production at 24 h than control cells, and *N*-acetylcysteine (NAC), a well-known antioxidant, attenuated this increase (Fig. 5h). Interestingly, NAC treatment suppressed AhR expression and its translocation into the nucleus induced by PM_2.5_ (Fig. 5i, j). Furthermore, NAC pretreatment of HaCaT and HEK001 cells and NHEKs reduced senescence, as evidenced by a decrease in the β-galactosidase activity induced by PM_2.5_ treatment (Fig. 5k). Finally, unlike control mice, PM_2.5_-treated mice showed hyperkeratotic epidermis in their skin tissue, a skin senescence phenomenon^29^ (Fig. 5l). The hyperkeratotic epidermis induced by PM_2.5_ occurred via increases in the expression levels of keratin-10, which is a differentiation marker, and PCNA, which is a proliferation marker (Fig. 5m). However, NAC pretreatment of PM_2.5_-treated mice reduced this condition (Fig. 5l, m). Thus, PM_2.5_ induces skin cellular senescence, at least in part, by activating the AhR–ROS signaling pathway.

### ROS are involved in the regulation of epigenetic modifiers by PM_2.5_

**Fig. 6:**
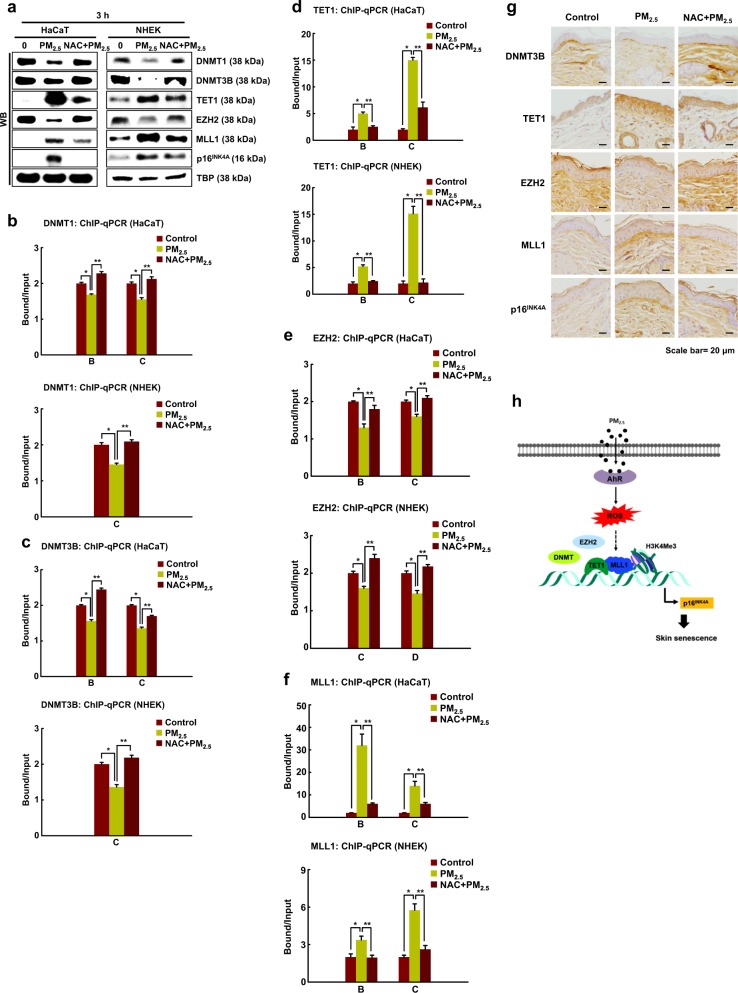
PM_2.5_-induced ROS regulate DNA and histone methylation status. **a** DNMT1, DNMT3B, TET1, EZH2, MLL1, and p16^INK4A^ were detected by western blotting with the corresponding antibodies. ChIP products using antibodies against **b** DNMT1, **c** DNMT3B, and **d** TET1, as well as **e** EZH2 and **f** MLL1, were analyzed by qPCR. **g** Skin sections were stained for DNMT3B, TET1, EZH2, MLL1, and p16^INK4A^ and counterstained with hematoxylin. *Significantly different from untreated cells (*p* < 0.05); **significantly different from PM_2.5_-treated cells (*p* < 0.05). **h** Working model for PM_2.5_-induced skin cellular senescence. In the presence of PM_2.5_, the PM_2.5_-AhR-ROS pathway prompts the binding of TET1 and MLL1 to the *p16*^*INK4A*^ locus in place of DNMT and EZH2. This binding induces p16^INK4A^ expression, which participates in the induction of skin cell senescence

We investigated whether ROS induction by PM_2.5_ affects cellular senescence-related epigenetic regulators. As shown in Fig. 6a, NAC pretreatment of PM_2.5_-treated cells reversed the changes in DNMT, DNMT3B, TET1, EZH2, and MLL1 expression and limited the induction of p16^INK4^ expression. These changes in the expression levels of these enzymes resulted in similar changes in their respective binding to the *p16*^*INK4*^ promoter, as measured by ChIP-qPCR (Fig. 6b–f). Finally, changes in the different epigenetic enzymes and the induction of p16 were also observed in the epidermal keratinocytes of mice treated with PM_2.5_, and importantly, NAC reversed these changes (Fig. 6g).

## Discussion

The skin is targeted directly by PM. PAHs present on the surface of PM cause harmful effects on the skin, including skin senescence, allergies, and inflammatory dermatitis. PM is known to induce the translocation of AhR to the nucleus, the activation of ERK and c-Jun, and the transcription of senescence-related proteins, such as CYP1A and MMP1^22^. Recently, we reported that HaCaT keratinocytes internalized PM_2.5_, induced oxidative stress^30^, and triggered inflammatory cytokine production via the activation of NF-κB by TLR5-NOX4-ROS signaling^21^. However, the underlying mechanism that regulates skin cell senescence induced by PAH-mediated oxidative stress has not been analyzed in terms of epigenetic control, including DNA methylation and histone modification. In the present study, we demonstrated that PAHs on PM_2.5_ induced human skin epidermal keratinocyte senescence through the expression of p16^INK4A^, a senescence inducer, and the induction of oxidative stress.

P16^INK4A^ is a specific CDK4/CDK6 inhibitor that plays a pivotal role in inducing cellular senescence. The p16^INK4A^ protein is relatively stable, and its expression is primarily regulated at the transcriptional level. Aberrant hypermethylation of the *p16*^*INK4A*^ gene is widely detected in most tumor types, leading to reduced gene expression^31–33^. In mammalian cells, DNMT1, DNMT3A, and DNMT3B participate in the maintenance of global DNA methylation and gene-specific de novo DNA methylation^34^. This methylation process can be reversed by DNA demethylases known as TETs. During PM_2.5_ exposure, TET replaces DNMT in the promoter region of *p16*^*INK4A*^, thereby promoting *p16*^*INK4A*^ transcription. In addition to DNA methylation, histone modification via the histone methyltransferases EZH2 and MLL1 is also crucial for gene transcription. The H3K27 methyltransferase EZH2 binds to the *p16*^*INK4A*^ locus, leading to the suppression of *p16*^*INK4A*^ transcription. Conversely, the binding of the H3K4 methyltransferase MLL1 to the *p16*^*INK4A*^ locus induces its transcription during replicative and premature senescence^35^. In the absence of PM_2.5_, EZH2 binds the proximal promoter (−0.3 to +85 bp) of *p16*^*INK4A*^ in HaCaT cells and a broader region (from +85 to +5.6 kb) in NHEKs, probably reflecting differential regulation between these cell lines. However, upon PM_2.5_ exposure, MLL1 replaced EZH2 in both cell lines, thereby promoting *p16*^*INK4A*^ transcription. Our previous studies showed that the DNA demethylase TET1 and the histone methyltransferase MLL1 interact to upregulate antioxidant transcription factors under oxidative stress^36^. This interaction also occurred upon PM_2.5_ exposure. Because MLL1- and TET1-binding sites overlap to a large extent with H3K4Me3 sites in the *p16*^*INK4A*^ promoter, PM_2.5_-induced interactions between these proteins might participate in the induction of *p16*^*INK4A*^ transcription.

ROS are known regulators of the methylation status of the *p16*^*INK4A*^ promoter via JNK-DNMT1 pathway activation in the lung epithelium and in myelodysplastic syndrome^37,38^. PM_2.5_ induces AhR expression and translocation, leading to ROS generation. ROS contribute to the induction and maintenance of the cellular senescence process, as evidenced by the delayed onset of replicative senescence when ROS production is counteracted through antioxidants^28^. PM_2.5_-induced toxic effects in humans involve oxidative stress mechanisms. PM_2.5_ has been shown to be associated with DNA damage, increased ROS formation, and accelerated cellular senescence^39^. We also demonstrated that the antioxidant NAC attenuated the senescence induced by PM_2.5_-generated ROS. NAC pretreatment of PM_2.5_-treated cells reversed the changes in DNMT, DNMT3B, TET1, EZH2, and MLL1 expression, thereby reversing the decreased p16^INK4^ expression and enabling cellular senescence escape.

To the best of our knowledge, this is the first report that skin senescence induced by the PM_2.5_–AhR–ROS pathway involves epigenetic regulation of the expression of the senescence-associated *p16*^*INK4*^ gene (Fig. 6h). These results may provide insights into the development of preventive and therapeutic strategies against skin senescence, which may help address skin aging in the current scenario of ever-increasing air pollution.

## References

[1] Son JY, Lee JT, Kim KH, Jung K, Bell ML (2012). Characterization of fine particulate matter and associations between particulate chemical constituents and mortality in Seoul, Korea. Environ. Health Perspect..

[2] Bartsch N, Heidler J, Vieth B, Hutzler C, Luch A (2016). Skin permeation of polycyclic aromatic hydrocarbons: a solvent-based in vitro approach to assess dermal exposures against benzo[a]pyrene and dibenzopyrenes. J. Occup. Environ. Hyg..

[3] Krutmann J (2014). Pollution and skin: from epidemiological and mechanistic studies to clinical implications. J. Dermatol. Sci..

[4] Pan TL (2015). The impact of urban particulate pollution on skin barrier function and the subsequent drug absorption. J. Dermatol. Sci..

[5] Qiao Y (2017). Airborne polycyclic aromatic hydrocarbons trigger human skin cells aging through aryl hydrocarbon receptor. Biochem. Biophys. Res. Commun..

[6] Abiko Y, Lin FY, Lee H, Puga A, Kumagai Y (2016). Quinone-mediated induction of cytochrome P450 1A1 in HepG2 cells through increased interaction of aryl hydrocarbon receptor with aryl hydrocarbon receptor nuclear translocator. J. Toxicol. Sci..

[7] Costa C (2010). Exposure of human skin to benzo[a]pyrene: role of CYP1A1 and aryl hydrocarbon receptor in oxidative stress generation. Toxicology.

[8] Birch-Machin MA, Bowman AA (2016). Oxidative stress and ageing. Br. J. Dermatol..

[9] Kim KE, Cho D, Park HJ (2016). Air pollution and skin diseases: adverse effects of airborne particulate matter on various skin diseases. Life Sci..

[10] Venza M (2015). Cellular mechanisms of oxidative stress and action in melanoma. Oxid. Med. Cell. Longev..

[11] Zhao L (2015). JMJD3 promotes SAHF formation in senescent WI38 cells by triggering an interplay between demethylation and phosphorylation of RB protein. Cell Death Differ..

[12] He S, Sharpless NE (2017). Senescence in health and disease. Cell.

[13] Baker DJ (2011). Clearance of p16Ink4a-positive senescent cells delays ageing-associated disorders. Nature.

[14] Ito S (2011). Tet proteins can convert 5-methylcytosine to 5-formylcytosine and 5-carboxylcytosine. Science.

[15] Zhao R, Choi BY, Lee MH, Bode AM, Dong Z (2016). Implications of genetic and epigenetic alterations of CDKN2A (p16(INK4a)) in cancer. EBioMedicine.

[16] Song W (2014). Increased p16 DNA methylation in mouse thymic lymphoma induced by irradiation. PLoS One.

[17] Zhu B (2017). Atorvastatin treatment modulates p16 promoter methylation to regulate p16 expression. FEBS J..

[18] D’Arcangelo D, Tinaburri L, Dellambra E (2017). The role of p16INK4a pathway in human epidermal stem cell self-renewal, aging and cancer. Int. J. Mol. Sci..

[19] Serra RW, Fang M, Park SM, Hutchinson L, Green MR (2014). A KRAS-directed transcriptional silencing pathway that mediates the CpG island methylator phenotype. Elife.

[20] Kotake Y, Zeng Y, Xiong Y (2009). DDB1-CUL4 and MLL1 mediate oncogene-induced p16INK4a activation. Cancer Res..

[21] Ryu YS (2019). Particulate matter induces inflammatory cytokine production via activation of NFκB by TLR5-NOX4-ROS signaling in human skin keratinocyte and mouse skin. Redox Biol..

[22] Lee CW (2016). Urban particulate matter down-regulates filaggrin via COX2 expression/PGE2 production leading to skin barrier dysfunction. Sci. Rep..

[23] Kia SK, Gorski MM, Giannakopoulos S, Verrijzer CP (2008). SWI/SNF mediates polycomb eviction and epigenetic reprogramming of the INK4b-ARF-INK4a locus. Mol. Cell. Biol..

[24] Herman JG (1996). a novel PCR assay for methylation status of CpG islands. Proc. Natl Acad. Sci. USA.

[25] McGarvey KM, Greene E, Fahrner JA, Jenuwein T, Baylin SB (2007). DNA methylation and complete transcriptional silencing of cancer genes persist after depletion of EZH2. Cancer Res..

[26] Aird KM, Zhang R (2013). Detection of senescence-associated heterochromatin foci (SAHF). Methods Mol. Biol..

[27] Zhou B (2017). Mitochondrial activity and oxidative stress functions are influenced by the activation of AhR-induced CYP1A1 overexpression in cardiomyocytes. Mol. Med. Rep..

[28] Davalli P, Mitic T, Caporali A, Lauriola A, D’Arca D (2016). ROS, cell senescence, and novel molecular mechanisms in aging and age-related diseases. Oxid. Med. Cell. Longev..

[29] Singh B, Schoeb TR, Bajpai P, Slominski A, Singh KK (2018). Reversing wrinkled skin and hair loss in mice by restoring mitochondrial function. Cell Death Dis..

[30] Piao MJ (2018). Particulate matter 2.5 damages skin cells by inducing oxidative stress, subcellular organelle dysfunction, and apoptosis. Arch. Toxicol..

[31] Emmett RA, Davidson KL, Gould NJ, Arasaradnam RP (2017). DNA methylation patterns in ulcerative colitis-associated cancer: a systematic review. Epigenomics.

[32] Han YD (2017). Associations of P16INK4a promoter hypermethylation with squamous intra-epithelial lesion, cervical cancer and their clinicopathological features: a meta-analysis. Oncotarget.

[33] Nikolic N (2015). High frequency of p16 and p14 promoter hypermethylation and marked telomere instability in salivary gland tumors. Arch. Oral. Biol..

[34] Zhou W (2016). Repeated PM2.5 exposure inhibits BEAS-2B cell P53 expression through ROS-Akt-DNMT3B pathway-mediated promoter hypermethylation. Oncotarget.

[35] Kotake Y, Naemura M, Murasaki C, Inoue Y, Okamoto H (2015). Transcriptional regulation of the p16 tumor suppressor gene. Anticancer Res..

[36] Kang KA (2016). Interaction of DNA demethylase and histone methyltransferase upregulates Nrf2 in 5-fluorouracil-resistant colon cancer cells. Oncotarget.

[37] Gonçalves AC (2016). Oxidative stress levels are correlated with P15 and P16 gene promoter methylation in myelodysplastic syndrome patients. Clin. Exp. Med..

[38] Soberanes S (2012). Particulate matter air pollution induces hypermethylation of the p16 promoter via a mitochondrial ROS-JNK-DNMT1 pathway. Sci. Rep..

[39] Gao ZX (2016). Assessment of DNA damage and cell senescence in corneal epithelial cells exposed to airborne particulate matter (PM2.5) collected in Guangzhou, China. Investig. Ophthalmol. Vis. Sci..

